# Canine Distemper Virus: Special Issue Editorial

**DOI:** 10.3390/v17121630

**Published:** 2025-12-17

**Authors:** Mónica G. Candela, Nieves Ortega

**Affiliations:** Animal Health Department, Faculty of Veterinary, University of Murcia, 30100 Murcia, Spain; nortega@um.es

This Special Issue of *Viruses* focuses on canine distemper virus (CDV) in a context where the disease continues to challenge clinical practice, epidemiology, and wildlife conservation, despite more than half a century of systematic vaccination. The eleven papers collected here provide an integrated view of CDV as a multi-host pathogen that is genetically diverse and can produce complex clinical pictures, while offering conceptual and methodological tools to improve its surveillance and control.

Canine distemper virus (CDV) remains a globally distributed, highly contagious morbillivirus that threatens domestic dogs and a broad range of wildlife species, despite decades of widespread vaccination [1,2]. Current evidence shows that CDV circulates in genetically diverse lineages with distinct geographical patterns, complicating molecular surveillance and raising concerns about vaccine escape in some populations [2,3,4]. At the same time, expanding host ranges and repeated spillover at the domestic–wildlife interface underscores the need for integrated One Health approaches that combine improved diagnostics, genomic monitoring, and context-adapted vaccination strategies [5,6,7].

Firstly, this Special Issue reinforces the notion that CDV is no longer a problem exclusive to domestic dogs but has become a cross-species risk for multiple orders of mammals. The study by Mares-Guia et al. describes the first natural co-infection with CDV and yellow fever virus in a neotropical primate of the genus Callithrix, highlighting both the plasticity of the virus and the importance of integrating morbillivirus surveillance into sentinel primate monitoring programmes [8]. Ríos-Usuga et al. show, for their part, the simultaneous circulation of the South America–North America 4 lineage in domestic dogs and crab-eating foxes (*Cerdocyon thous*) in an Andean valley, evidencing the existence of a genuine domestic–wildlife interface in which wild canids act as an epidemiological bridge and potential wildlife reservoir [9]. Rendón-Marín et al. extend this perspective by documenting infections in other wild and peri-urban carnivores, reinforcing the idea of ecologically complex metareservoirs where the elimination of the virus in a single species is insufficient to interrupt its circulation [10].

In the field of clinical practice and epidemiology, the articles in this Special Issue specifically address the neurological form and individual determinants of the disease. Oliver-Guimerà et al. deepen the clinical and neuropathological characterisation of distemper encephalomyelitis, highlighting the high frequency of multifocal lesions, the prognostic role of epileptic seizures, and the need for a specialised neurological approach to improve the survival and quality of life of affected animals [11]. Freire et al. analyse a large cohort of dogs with neurological signs and demonstrate that young animals, particularly those of the Shih Tzu and Lhasa Apso breeds, have a significantly increased risk of developing neurological manifestations associated with CDV, with marked seasonality in autumn and a mortality rate of nearly half of confirmed cases [12].

A long-recognised gap in the management of distemper concerns sensitive diagnosis in the early stages and in animals with low viral load or chronic symptoms. Iribarnegaray et al. compare conventional RT-PCR, RT-qPCR, and digital PCR in droplets (ddPCR), demonstrating that the third achieves detection limits of just a few copies per reaction volume, with the highest sensitivity and concordance with clinical diagnosis among the techniques evaluated, positioning it as a reference tool for confirming doubtful cases and studying subclinical carriers [13]. Verdes et al. address the optimisation of sampling and the selection of tissues and fluids at different stages of infection, providing practical criteria for maximising the diagnostic performance of molecular and immunohistochemical techniques in routine laboratory practice [14]. My-Van et al., from a more applied perspective, evaluate diagnostic algorithms that combine rapid antigen tests, RT-qPCR, and serological tests in different clinical scenarios, proposing tiered strategies that allow diagnostic efforts to be adapted to the available resources without sacrificing accuracy in critical cases [15].

Another critical aspect that this Special Issue helps to clarify is the relationship between genetic variability, vaccine strains, and protection failures. Candela et al. present a comprehensive global review of the spatial and functional dispersion of the America-1 and Rockborn-like vaccine genotypes, documenting cases of post-vaccination disease and detection of vaccine-derived strains in multiple domestic, synanthropic, and wild species and introducing the concept of the “animalisation” of vaccine strains, i.e., their adaptation and sustained circulation in host populations [16]. Verdes et al. and Gutiérrez et al. provide field data showing the emergence of clinical cases in dogs with up-to-date vaccination schedules, raising essential questions about antigenic mismatches between vaccine strains and circulating lineages, as well as problems in the cold chain and the handling and administration of biologicals [14,17]. Oğuzoğlu et al. perform a detailed phylogenetic and functional analysis of field strains from different lineages, identifying mutations in key genes such as *H* and *Fsp* that could be associated with increased neurovirulence, species jumping, and possible decreased neutralisation by antibodies induced by classical vaccines [18].

From the perspective of veterinary public health and conservation, this Special Issue also offers valuable conceptual frameworks for interpreting CDV circulation in complex ecosystems. Ríos-Usuga et al. show that the South America–North America-4 lineage is repeatedly introduced into the same valley through combinations of wildlife movements and domestic dog movements, supporting the idea of multiple waves of reintroduction rather than a single sustained chain of transmission [9]. Rendón-Marín et al. and Mares-Guia et al. illustrate how synanthropic mesocarnivores and neotropical primates can play bridging and sentinel roles, respectively, with direct implications for the design of integrated One Health surveillance programmes [8,10]. Candela et al. incorporate ecological and behavioural variables of the hosts to propose conceptual models of the spread of vaccine-derived strains along urban–peri-urban–natural gradients, emphasising the central role of dogs and synanthropic mesocarnivores as connectors between epidemiological compartments [16]. Gutierrez et al. complement these findings with epizootiological data from canine populations in different geographical and socioeconomic contexts, highlighting how population density, unequal access to vaccinations, and interaction with wildlife influence the persistence of CDV in urban and rural areas [17].

Together, the eleven articles in this Special Issue have addressed key gaps in knowledge about CDV: its ability to infect new hosts and co-infect with other viruses relevant to human and animal health [8,10], the detailed characterisation of the neurological form and its risk factors [11,12], the improvement of diagnostic tools using ddPCR and integrated algorithms [13,14,15], and understanding the dynamics of lineages, including those related to vaccines, in a network of domestic and wild hosts [9,16,17,18]. Far from closing the debate, these studies instead highlight priorities for future research: deploying integrated, wide-ranging genomic surveillance programmes; redefining vaccination strategies considering lineage diversity and episodes of apparent failure; strengthening diagnostic capacity with highly sensitive technologies; and promoting interdisciplinary studies involving virology, ecology, clinical neurology, and conservation science.

As we conclude our work on this first Special Issue of *Viruses* on canine distemper virus, the central lesson is that CDV remains an ideal model for emerging and re-emerging pathogens at the intersection of animal health, biodiversity, and the human–domestic-wildlife interface. The scientific community now has new data, tools, and conceptual frameworks provided by Mares-Guia et al. [8], Rios-Usuga et al. [9], Rendón-Marín et al. [10], Guimerà et al. [11], Freire et al. [12], Iribarnegaray et al. [13], Verdes et al. [14], Van et al. [15], Candela et al. [16], Gutiérrez et al. [17], and Oğuzoğlu et al. [18] to move towards more effective and sustainable control strategies. The key challenge in the coming years will be to translate this knowledge into better-tailored vaccination policies, truly integrated surveillance systems, and conservation actions to reduce the impact of CDV on domestic and wild species through a One Health approach. We hope that the following in-depth analyses of new epidemiological trends in CDV, contributions regarding the control and study of carriers at the human–domestic–wildlife interface, and advances in the diagnosis and histopathology of this virus will be of interest to researchers and readers of the journal *Viruses*.
